# Impact of legalization on cannabis exposure calls to the British Columbia Poison Control Centre

**DOI:** 10.17269/s41997-025-01022-8

**Published:** 2025-04-07

**Authors:** Jeffrey Trieu, Nina Dobbin, Sarah B. Henderson, David McVea

**Affiliations:** 1 Environmental Health Services, British Columbia Centre for Disease Control, Vancouver, BC Canada; 2 National Collaborating Centre for Environmental Health, Vancouver, BC Canada; 3 School of Population and Public Health, University of British Columbia, Vancouver, BC Canada

**Keywords:** Cannabis, Marijuana, Poison control centres, Interrupted time series analysis, Cannabis, Marijuana, Centres antipoison, Analyse de série chronologique interrompue

## Abstract

**Objective:**

The objective of this study was to examine whether cannabis exposure calls to the British Columbia Drug and Poison Information Centre (DPIC) were impacted by the legalization of non-medical cannabis in Canada.

**Methods:**

We fit interrupted time series models to monthly counts of cannabis cases from 2013 to 2021, stratified by age and cannabis form. We set the intervention month to October 2018 legalization for cases involving inhaled dried cannabis and ingestible oils and capsules. We set the intervention month to January 2020 for cases involving edibles and inhaled concentrates to reflect their commercial rollout after their October 2019 legalization.

**Results:**

DPIC managed 3989 cases involving cannabis exposure between 2013 and 2021. The rate (95% CI) of all cannabis cases increased by 17% (14%, 20%) annually from 2013 to October 2018 legalization. The highest pre-legalization increase was in pediatric edible cases with 52% (36%, 69%) and 57% (35%, 82%) annual increases among children aged 5 and under and 6 to 12, respectively. Upon legalization, the rate of cases consuming oil and capsule products spiked by 26% (− 19%, 96%) followed by a decrease, but remaining higher than the pre-legalization rate. Legalization did not have an immediate effect on the rate of cases involving edibles or inhaled cannabis, which all continued to increase post-legalization, albeit at slower rates.

**Conclusion:**

Regardless of the contributing factors to cannabis case trends at DPIC, these data highlight the importance of poisoning prevention policies, promotion of low-risk use, and routine surveillance.

**Supplementary Information:**

The online version contains supplementary material available at 10.17269/s41997-025-01022-8.

## Résumé

**Objectif:**

Déterminer si la légalisation du cannabis à des fins non médicales au Canada a entraîné des répercussions sur les appels au centre antipoison (DPIC) de la Colombie-Britannique en raison d’expositions au cannabis.

**Méthode:**

Nous avons jumelé des modèles de séries chronologiques interrompues aux chiffres mensuels sur les cas de cannabis entre 2013 et 2021, stratifiés selon l’âge et la forme de cannabis. Pour les cas impliquant du cannabis séché inhalé et des huiles et capsules ingérables, nous avons fixé le mois d’intervention à octobre 2018 (le mois de leur légalisation). Pour les cas impliquant des produits comestibles et des concentrés inhalés, nous avons fixé le mois d’intervention à janvier 2020 (le mois de leur sortie commerciale après leur légalisation en octobre 2019).

**Résultats:**

Le DPIC a géré 3989 cas impliquant des expositions au cannabis entre 2013 et 2021. Le taux (IC de 95%) de l’ensemble des cas de cannabis a augmenté de 17% (14%, 20%) par année entre 2013 et la légalisation en octobre 2018. Avant la légalisation, la hausse la plus prononcée concernait les cas d’ingestion de produits comestibles par des enfants, qui ont augmenté annuellement de 52% (36%, 69%) chez les enfants de 5 ans et moins et de 57% (35%, 82%) chez les enfants de 6 à 12 ans. Après la légalisation, le taux des cas de consommation d’huiles et de capsules a grimpé de 26% (-19%, 96%) pour diminuer par la suite, mais il est resté plus élevé que le taux d’avant la légalisation. La légalisation n’a pas eu d’effet immédiat sur le taux des cas impliquant des produits comestibles ou du cannabis inhalé, lesquels ont continué d’augmenter après la légalisation, bien que plus lentement.

**Conclusion:**

Indépendamment des facteurs qui contribuent aux tendances dans les cas de cannabis au DPIC, ces données montrent l’importance des politiques de prévention des empoisonnements, de la promotion de la consommation à faible risque et de la surveillance de routine.

## Introduction

In 2015, Canadians elected a majority government that campaigned on the decriminalization, legalization, and regulation of non-medical cannabis use, possession, and sale. Following a multi-year consultation and planning process, the Cannabis Act came into effect on October 17, 2018. Initially, this legalized and regulated four activities: (i) the provincially licensed sale of dried or fresh cannabis flower and cannabis oil products; (ii) possession of up to 30 g of dried cannabis or an equivalent quantity in a non-dried form; (iii) growth of up to four cannabis plants per residence for personal use; and (iv) preparation of cannabis products, such as edibles, for personal use (Government of Canada, 2018). One year later, the Cannabis Act allowed for the provincially licensed sale of edibles, concentrates, and topicals. In British Columbia (BC), the commercial rollout of these products was delayed until late December 2019 to early 2020 (Government of British Columbia, 2019).

Several studies and surveys have examined how the legalization of cannabis products has impacted cannabis use and population health. The National Cannabis Survey found that since initial legalization in 2018, the prevalence of cannabis use has increased, more individuals are acquiring cannabis from legal sources, and edibles are more widely used (Government of Canada, 2021b). Myran et al. (2023a) found that the legalized retail of edibles, concentrates, and topicals in 2019 and their commercial rollout in early 2020 was associated with increased rates of cannabis-related hospitalizations in Ontario, Quebec, Alberta, and BC among those age 15 and older, which was not observed after initial legalization in 2018. The specific conditions with the highest relative increases were cannabis-induced psychosis and cannabis withdrawal. Given the overlap in timing, the authors noted that it was difficult to disentangle the effects of increased cannabis availability from legalized retail from increased risky cannabis use associated with the COVID-19 pandemic. A similar study found that pediatric cannabis–related hospitalizations significantly increased in Ontario, Alberta, and BC in early 2020, where the sale of edibles was permitted, but not in Quebec where it was largely prohibited (Myran et al., 2023b). Overall, there has been an expected increase in cannabis use among Canadians given its increased social acceptability and accessibility with legalization. With this has come a rise in cannabis-related health harms that range from unintentional pediatric poisonings to disorders related to heavy use (Hall et al., 2023). It is important to continue investigating patterns in cannabis use and its harms in order to inform policies and practices in the current regulatory framework.

Poison centres provide another valuable source of data to examine population patterns in cannabis use and associated harms (Allaf et al., 2023). Poison centre data also allow for the surveillance of events that do not require further medical attention and therefore would not be captured by other surveillance systems that use administrative health data. Cannabis exposure cases tripled from 2013 to 2018 at the BC Drug and Poison Information Centre (DPIC), one of five Canadian poison centres (Rahim, 2019). In this study, we use the detailed information available through DPIC data to describe cannabis exposure events by cannabis form, use history, and exposure intentionality, in addition to age and sex. We also implement an interrupted time series analysis to examine the impact of legalization on specific forms of cannabis exposures reported to DPIC between 2013 and 2021.

## Methods

### Setting and data source

Poison centres are staffed by medical toxicologists, as well as nurses and pharmacists who are additionally certified as specialists in poison information (SPIs). DPIC SPIs operate a 24/7 phone service that receives calls from the public as well as health care professionals, and provide toxicological support and clinical guidance to callers. SPIs document case information in an electronic medical records system using standardized data fields consistent with the American Poison Centers (APC) National Poison Data System (NPDS) (Durigon et al., 2013). SPIs also record a case note in a free-text narrative format, which includes pertinent patient details, information about the exposure, the recommendations given, and resolution of the case.

**Fig. 1:**
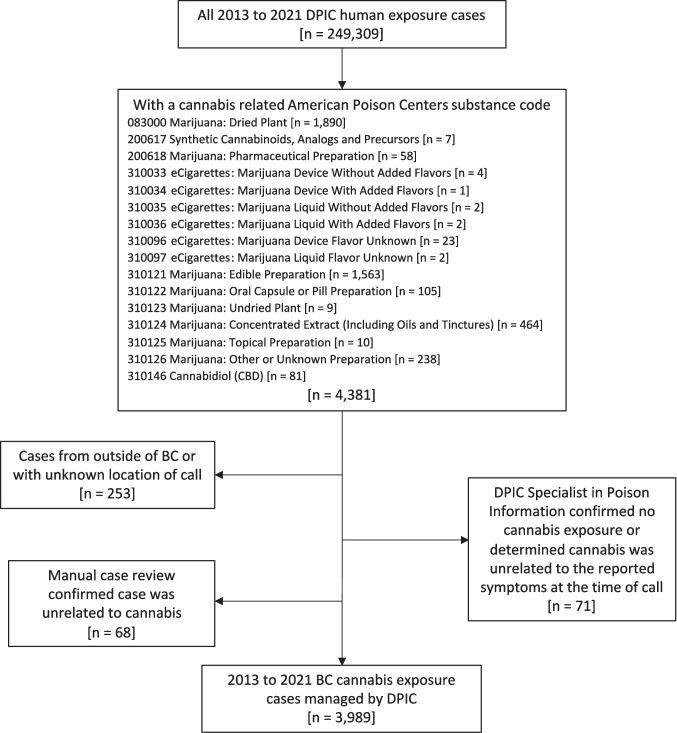
Drug and Poison Information Centre (DPIC) case inclusion and exclusion flow chart. Substance code–specific case counts sum to greater than total due to cases with more than one cannabis-related substance code

We included BC cases that had an exposure to cannabis as defined by an APC substance code (Fig. 1). We reviewed all case notes and removed cases that were erroneously coded as a cannabis exposure. We also removed cases where the SPI confirmed there was no cannabis exposure or judged the clinical effects to be unrelated to cannabis. We included cases between 2013, the first year electronic records from DPIC were available, and 2021, the last year for which we had completed data validation and extraction.

Trained case reviewers validated and extracted data from DPIC case notes (Table 1). One trained reviewer validated SPI-documented age, sex, caller identity, patient flow, and clinical outcome against the case note for the file. We used the information in the case note in the event of incongruence because its free-text nature provides more contextual information. One trained reviewer extracted cannabis use history and cannabis form as they were stated verbatim in the case note. Two trained reviewers extracted exposure intentionality from the case note which, unlike the other case-reviewed variables, required some subjective interpretation. A third trained reviewer resolved any disagreements between the initial two reviewers.

**Table 1:** Data fields used for the analysis, collected by specialists in poison information (SPI) or extracted from review of the free-text case notes. All data fields include an unknown category

Data field	Categories	Definition
Age group	• 5 years and under • 6 to 12 years • 13 to 18 years • 19 to 29 years • 30 + years	Age of the patient as volunteered from the caller, documented by the SPI and validated against the SPI documented free-text case note
Sex	• Female • Male	Sex of the patient as volunteered from the caller, documented by the SPI and validated against the SPI documented free-text case note
Patient flow	• Already in or on the way to a health care facility • Managed without further medical attention • Referred to a health care facility	Categorical description of the patient’s contact with the health care system at the time of call to DPIC, as documented by the SPI and validated against the SPI documented free-text case note
Caller identity	• Family or friend • Self • Health care professional • Other	Categorical description of the relationship of the caller to the patient, as documented by the SPI and validated against the SPI documented free-text case note
Clinical outcome	• No effect • Minor effect • Moderate effect • Major effect • Unable to follow, but potentially toxic exposure	Categorical description of the patient’s clinical outcome, as documented by the SPI and validated against the SPI documented free-text case note. We did not use clinical judgement in these case reviews and only made corrections if there was a clear mis-entry Minor effect is defined as involving symptoms that are minimally bothersome to the individual. Moderate effect is defined as involving more pronounced or prolonged symptoms, usually requiring some form of treatment, but with no risk of death or disability. Major effect is defined as involving symptoms that risk death or disability. Unable to follow, but potentially toxic exposure is defined as a patient lost to follow-up but the exposure suggests moderate or major clinical effects are possible
Cannabis use history	• New or inexperienced user • Previous user	Variable derived from review of the free-text case notes. We assumed that all individuals aged 12 and under were new users unless explicitly stated otherwise
Cannabis substance form	• Edibles • Inhaled dried flower • Inhaled concentrates • Ingestible oils and capsules • Ingestion of dried cannabis or paraphernalia • Pharmaceutical cannabinoids • Second-hand cannabis smoke • Synthetic cannabinoids • Topicals • Other	Variable derived from review of the free-text case notes. We defined edibles as ingested cannabinoid–infused food products. We defined ingestible oils and capsules as non-food oils and non-pharmaceutical capsules
Exposure intentionality	• Intentional self-harm use • Recreational • Therapeutic intent • Unintentional use	We defined unintentional use or intentional self-harm use as documented by the SPI. Self-harm use is defined as an exposure resulting from the inappropriate use of a substance for self-harm or for self-destructive or manipulative reasons. We defined a case as therapeutic use or recreational use from review of the free-text case notes. We defined a case as therapeutic use if the free-text case notes mentioned treatment of a medical condition or use to improve general well-being, such as aiding sleep. We defined cases as recreational use if contextual information in the case notes suggested such (e.g., at a party) or if the use was not therapeutic, unintentional, or for self-harm, and cannabis was used on its own, with other commonly used recreational substances such as alcohol, or with routine medications not taken for recreational use

### Statistical analysis

To evaluate the effect of cannabis legalization on the time trend of cases, we fit interrupted time series models with a Poisson distribution to monthly counts of all cases and for the following cannabis form strata: inhaled dried cannabis, inhaled concentrates, ingestible oils and capsules, and edibles. For the model including all cases, the inhaled dried cannabis model, and ingestible oil and capsule model, we defined October 2018 as the intervention month to align with the enactment of the BC Cannabis Act. This legislative step legalized the possession and use of non-medical cannabis and legalized the regulated sale of these specific cannabis products. For the edible and inhaled concentrate models, we defined January 2020 as the intervention month, when commercial rollout of these legalized products approximately began in BC. To evaluate the sensitivity of the chosen intervention month on the fitted time trends, we also fit models with the intervention month set as the preceding and subsequent 3 months. In addition, we fit age-stratified models by the following age groups when there were at least 100 total cases: 5 years and under, 6 to 12 years, 13 to 18 years, 19 to 29 years, and 30 years and older. We used annual age-specific provincial population estimates as the model offset (Government of British Columbia, 2021). We interpreted the pre- and post-intervention time trends as annual percentage change in the rate and the intervention effect as the percentage change of the rate from pre-intervention level and slope. When statistically significant overdispersion was found in a Poisson model (Gelman & Hill, 2007), we fit a negative binomial model instead. We checked for seasonality by examining boxplots and running Kruskal–Wallis tests by month and annual quarter. We did not find any statistically significant differences by month or quarter, therefore did not account for any seasonal trends in the interrupted time series models (Supplementary Material 1). Whenever we computed a single case rate across the study period, we used provincial population estimates from 2017, the median year of the study period, and computed 95% confidence intervals using Poisson exact tests. We completed all data preparation and analysis in the R statistical computing environment version 4.3.2 (R Core Team, 2021).

## Results

### Case summary

From 2013 to 2021, DPIC managed 3989 cases involving cannabis exposure in British Columbia (Fig. 1; Table 2). Age-specific case rates per 100,000 were highest in teens aged 13 to 18 (209.9), followed by children aged 5 and under (142.5), and young adults aged 19 to 29 (130.5). Children aged 12 and under were more likely to be referred to a health care facility by DPIC or already at or on their way to a health provider at the time of call. In contrast, teens and adults were more likely to not be referred for further medical attention (Table 3). Cases involved a wide variety of cannabis forms. The most common form of cannabis was edibles, which were involved in 1686 cases (42%). Inhalation was the second most common route of exposure, with 987 cases (25%) involving dried cannabis flower and 121 cases (3%) involving a concentrate. A total of 594 cases (15%) involved exposure to an ingestible cannabis oil or capsule product. There were several other less frequent forms of cannabis exposure. For example, 74 cases (2%) involved a pharmaceutical cannabinoid such as nabilone, 71 cases (2%) involved consumption of dried cannabis or some form of cannabis paraphernalia, and 66 cases (2%) involved second-hand smoke exposure. The form of cannabis was unknown in 418 cases (10%).

**Table 2:** Frequencies and percentages of case characteristics

	Case count (%)
Total cases	3898
Sex	
Male	2014 (50%)
Female	1958 (49%)
Unknown	17 (0%)
Polysubstance indicator	
Cannabis exposure only	2871 (72%)
Cannabis polysubstance exposure	1118 (28%)
Substance form^a^	
Edibles	1686 (42%)
Inhaled dried flower	987 (25%)
Ingestible oils and capsules	594 (15%)
Inhaled concentrates	121 (3%)
Pharmaceutical cannabinoids	74 (2%)
Ingestion of dried cannabis or paraphernalia	71 (2%)
Second-hand cannabis smoke	66 (2%)
Topicals	18 (0.5%)
Synthetic cannabinoids	7 (0.2%)
Other	6 (0.2%)
Unknown	418 (10%)
Caller identity	
Family or friend	1706 (43%)
Self	1142 (29%)
Health care professional	922 (23%)
Other	83 (2%)
Unknown	136 (3%)
Patient flow	
Managed without further medical attention	2392 (60%)
Already in or on the way to a health care facility	903 (23%)
Referred to a health care facility	602 (15%)
Unknown	92 (2%)
Exposure intentionality	
Recreational use	2326 (58%)
Unintentional exposure	929 (23%)
Therapeutic intent	356 (9%)
Intentional self-harm use	238 (6%)
Unknown	140 (4%)
Clinical outcome	
No effect	106 (3%)
Minor effect	2295 (58%)
Moderate effect	1214 (30%)
Major effect	99 (2%)
Unable to follow — potentially toxic exposure	275 (7%)
Cannabis use history	
Previous user	1210 (30%)
New/inexperienced user	1141 (29%)
Unknown	1638 (41%)

^a^Some cases involve multiple forms of cannabis exposure, so counts sum to greater than the total number of cases

**Table 3:** Age- and sex-specific case rates per 100,000 persons and age-specific case rates per 100,000 persons by cannabis substance form and patient flow. 95% confidence intervals from Poisson exact tests are in square brackets. The non-age-stratified case rates include 576 cases with unknown age

	All ages	≤ 5 years	6 to 12 years	13 to 18 years	19 to 29 years	30 + years
All cases	80.8 [78.4, 83.4]	142.5 [128.8, 157.2]	50.2 [42.9, 58.3]	209.9 [194.0, 226.7]	130.5 [122.4, 139]	39.9 [37.7, 42.1]
Sex						
Male cases	82.4 [78.9, 86.1]	151.7 [132.3, 173.3]	55.8 [45.2, 68.1]	212.7 [190.6, 236.6]	126.1 [115.1, 137.9]	40.7 [37.6–44.0]
Female cases	78.6 [75.2, 82.2]	131.2 [112.5–152.0]	44.4 [34.8, 55.8]	206.3 [184.0, 230.6]	135.2 [123.4, 147.8]	39.1 [36.2, 42.2]
Substance form						
Cannabis edibles	34.2 [32.6, 35.8]	91.5 [80.6, 103.5]	44.2 [37.4, 51.9]	55.0 [47.0, 63.9]	51.8 [46.8, 57.3]	16.6 [15.2–18.0]
Inhaled dried cannabis	20.0 [18.8, 21.3]	0 [0, 1.3]	1.8 [0.7, 3.9]	100.6 [89.7, 112.4]	44.0 [39.3–49.0]	6.2 [5.4, 7.1]
Ingestible oils and capsules	12.0 [11.1–13.0]	11.5 [7.9, 16.2]	2.4 [1.0, 4.7]	8.1 [5.2, 11.9]	14.1 [11.5, 17.1]	10.6 [9.5, 11.7]
Inhaled concentrates	2.5 [2.0, 2.9]	4.7 [2.5–8.0]	0.3 [0, 1.7]	10.0 [6.8, 14.2]	5.6 [4.0, 7.5]	0.6 [0.4, 0.9]
Pharmaceutical cannabinoids	1.5 [1.2, 1.9]	1.4 [0.4, 3.7]	0.3 [0, 1.7]	1.0 [0.2, 2.8]	1.5 [0.7, 2.7]	1.3 [1.0, 1.8]
Ingestion of dried cannabis or paraphernalia	1.4 [1.1, 1.8]	18.3 [13.6, 24.1]	0 [0, 1.1]	0 [0, 1.2]	0.7 [0.2, 1.6]	0.3 [0.1, 0.6]
Second-hand cannabis smoke	1.3 [1.0, 1.7]	5.0 [2.7, 8.4]	0 [0, 1.1]	2.3 [0.9, 4.7]	1.4 [0.7, 2.5]	0.4 [0.2, 0.7]
Topicals	0.4 [0.2, 0.6]	2.2 [0.8, 4.7]	0 [0, 1.1]	0 [0, 1.2]	0.1 [0, 0.8]	0.2 [0.1, 0.5]
Synthetic cannabinoids	0.1 [0.1, 0.3]	0 [0, 1.3]	0 [0, 1.1]	0.6 [0.1, 2.3]	0.1 [0, 0.8]	0.1 [0, 0.2]
Patient flow						
Managed without further medical attention	48.5 [46.6, 50.5]	29.8 [23.7, 36.9]	8.9 [6.0, 12.7]	112.2 [100.7, 124.7]	89 [82.3, 96.1]	26.9 [25.2, 28.8]
Referred to a health care facility	12.2 [11.2, 13.2]	55.3 [46.9, 64.7]	18.7 [14.4, 23.9]	25.5 [20.2, 31.8]	14.4 [11.8, 17.4]	4.3 [3.6–5.0]
Already in or on the way to a health care facility	18.3 [17.1, 19.5]	57.4 [48.9, 67]	22.6 [17.8, 28.2]	67.6 [58.7, 77.4]	22.9 [19.6, 26.7]	7.6 [6.7, 8.6]
Caller type						
Family or friend	34.6 [33.0, 36.3]	85.4 [74.9–97.0]	24.9 [19.9, 30.9]	92.2 [81.8, 103.5]	59.3 [53.9, 65.1]	16.4 [15.1, 17.9]
Self	23.1 [21.8, 24.5]	0.4 [0–2.0]	0.3 [0, 1.7]	25.2 [19.9, 31.5]	42.3 [37.8, 47.3]	13.2 [12.0, 14.5]
Health care professional	18.7 [17.5, 19.9]	52.8 [44.6–62.0]	21.7 [17.0, 27.3]	71.1 [62.1, 81.2]	22.7 [19.3, 26.4]	8.4 [7.5, 9.5]

Cannabis form trends differed by age and exposure intentionality (Table 4). The highest rate per 100,000 was for unintentional use of cannabis edibles among children aged 5 and under (90.8), followed by recreational use of inhaled dry cannabis among teens aged 13 to 18 (89.3). Other elevated rates were for recreational use of edibles among teens (45.0) and young adults (44.9), unintentional use of edibles among children 6 to 12 years (41.6), and recreational use of dried cannabis among young adults aged 19 to 29 (39.7).

**Table 4:** Age-specific case rates per 100,000 persons by cannabis substance form and exposure intentionality. 95% confidence intervals from Poisson exact tests are in square brackets

	Recreational	Unintentional use	Therapeutic intent	Intentional self-harm use	Unknown
Cannabis edibles					
5 years and under	0	90.8 [80.0, 102.7]	0.7 [0.1, 2.6]	0	0
6 to 12 years	1.2 [0.3–3.0]	41.6 [35.0, 49.1]	0.3 [0, 1.7]	0	1.2 [0.3–3.0]
13 to 18 years	45 [37.8, 53.1]	5.2 [3.0, 8.4]	1 [0.2, 2.8]	1.6 [0.5, 3.8]	2.3 [0.9, 4.7]
19 to 29 years	44.9 [40.2–50.0]	3.9 [2.6, 5.6]	1.1 [0.5, 2.1]	0.7 [0.2, 1.6]	1.2 [0.6, 2.3]
30 + years	10.8 [9.7, 12]	3 [2.5, 3.7]	2.1 [1.7, 2.7]	0.2 [0.1, 0.5]	0.3 [0.2, 0.6]
Inhaled dried cannabis					
5 years and under	0	0	0	0	0
6 to 12 years	1.5 [0.5, 3.5]	0	0	0	0.3 [0, 1.7]
13 to 18 years	89.3 [79.0, 100.4]	0	0.6 [0.1, 2.3]	9.4 [6.3, 13.5]	1.3 [0.4, 3.3]
19 to 29 years	39.7 [35.3, 44.6]	0	1.1 [0.5, 2.1]	2.6 [1.6–4.0]	0.5 [0.1, 1.4]
30 + years	5.1 [4.3, 5.9]	0	0.6 [0.4–1.0]	0.3 [0.2, 0.6]	0.1 [0, 0.3]
Ingestible oils and capsules					
5 years and under	0	11.1 [7.6, 15.8]	0.4 [0–2.0]	0	0
6 to 12 years	0.3 [0, 1.7]	1.8 [0.7, 3.9]	0	0.3 [0, 1.7]	0
13 to 18 years	5.5 [3.2, 8.8]	1.0 [0.2, 2.8]	0.3 [0, 1.8]	1.3 [0.4, 3.3]	0
19 to 29 years	9.9 [7.8, 12.5]	0.9 [0.4–2.0]	2.3 [1.3, 3.7]	0.3 [0–1.0]	0.7 [0.2, 1.6]
30 + years	3.5 [2.9, 4.3]	2.4 [1.9–3.0]	4 [3.3, 4.8]	0.1 [0, 0.3]	0.5 [0.3, 0.8]
Inhaled concentrates					
5 years and under	0	4.7 [2.5–8.0]	0	0	0
6 to 12 years	0	0.3 [0, 1.7]	0	0	0
13 to 18 years	9.7 [6.5, 13.8]	0	0	0.3 [0, 1.8]	0
19 to 29 years	5.3 [3.8, 7.2]	0	0.1 [0, 0.8]	0	0.1 [0, 0.8]
30 + years	0.4 [0.2, 0.7]	0	0.1 [0, 0.2]	0	0.1 [0, 0.2]

### Use history

Among cases aged 13 and older, cannabis use history was unknown for 48% (*n* = 1386) of cases. A total of 1021 cases (35%) were previous users and 518 cases (18%) were new or inexperienced users. Among new or inexperienced users aged 13 and up, most did not involve use of other substances (*n* = 472; 91%), were not referred for further medical attention after their call to DPIC (*n* = 416; 77%), involved consumption of an edible cannabis product (*n* = 321; 62%), and tried cannabis for recreational purposes (*n* = 364; 70%).

### Exposure intentionality

A total of 238 cases (6%) involved cannabis use with self-harm intent. Nearly all these cases involved cannabis use with other substances (*n* = 224; 94%). Among these, cannabis was usually not ranked as the most clinically relevant exposure (*n* = 216; 91%). All cases of use with self-harm intent were already at or on their way to a health care facility when the call to DPIC was made, or they were referred to a health care facility by DPIC. Three hundred fifty-six cases (9%) stated they used cannabis for a therapeutic benefit. These cases were nearly all adults aged 19 and over (*n* = 280; 79%) or had unknown age (*n* = 66; 19%). The majority were not referred for further medical attention after guidance from DPIC (*n* = 274; 77%).

A total of 929 cases (23%) were unintentional exposures: 545 cases (59%) were among children aged 12 and under and 303 cases (33%) were among teens and adults aged 13 and older. Age was unknown in 81 cases (9%). The cases among children aged 12 and under were primarily due to pediatric consumption of an edible (*n* = 393; 72%). Ninety-three pediatric cases (17%) were due to consumption of an ingestible oil product, pharmaceutical or non-pharmaceutical capsule, dried cannabis itself, or paraphernalia. Most of these pediatric consumption cases required some level of medical care. A total of 224 cases (41%) were already at or on their way to a health care facility when the call to DPIC was made and 216 cases (40%) were referred to a health care facility by DPIC. Forty-eight percent of the teen and adult cases (*n* = 144) were due to unintentional consumption of an edible and 36% of cases (*n* = 109) were due to the unintentional consumption of an ingestible oil product, pharmaceutical or non-pharmaceutical capsule, dried cannabis itself, or paraphernalia. In contrast to pediatric exposures, most teens and young adults were not referred for further medical attention after guidance from DPIC (*n* = 223; 74%).

### Pre-legalization time trends

**Fig. 2:**
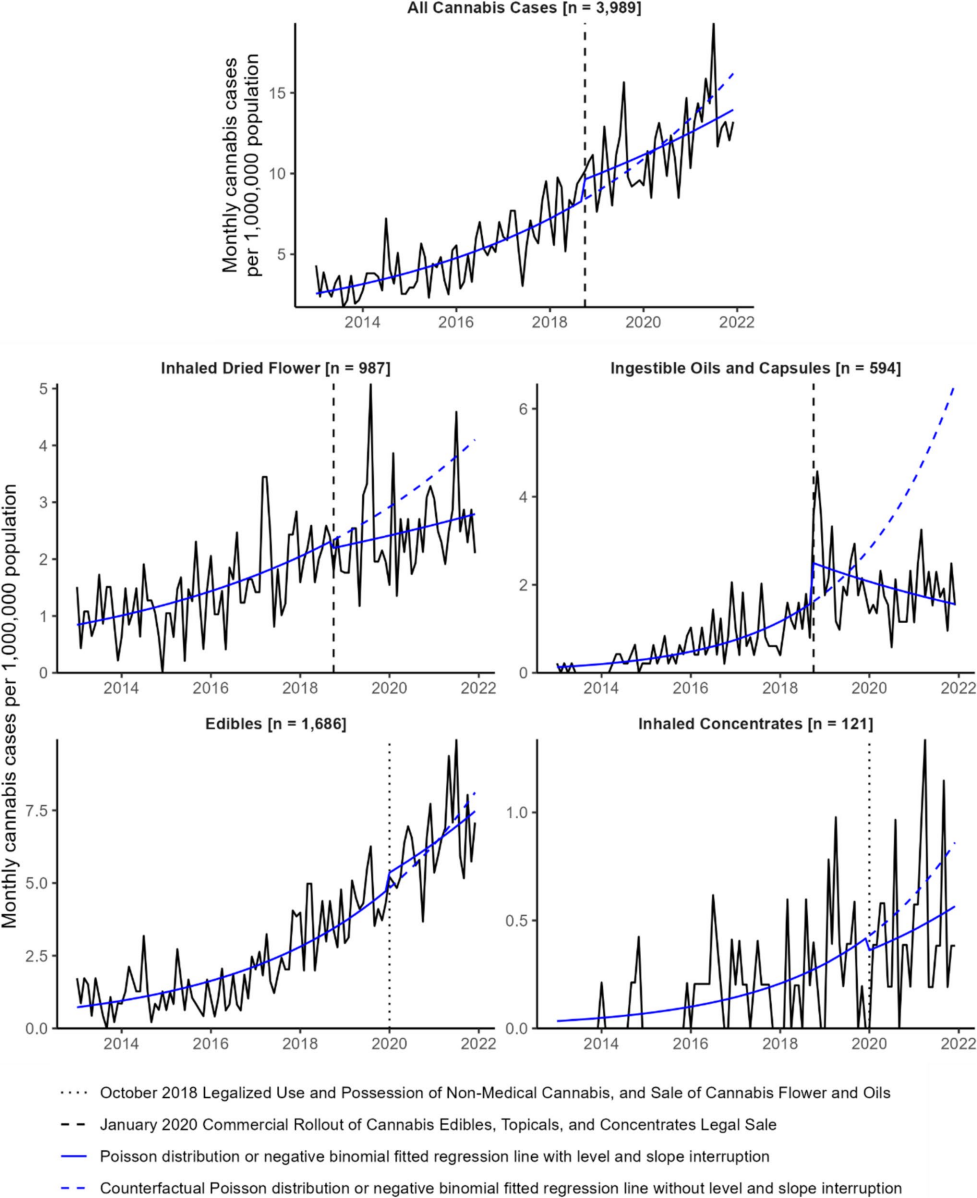
Monthly case rates per 1,000,000 population for all cannabis exposures and by substance form from 2013 through 2021. Negative binomial or Poisson distribution fitted regression lines were fit to each substance form–specific time series with a level and slope intervention at their respective month of legalized sale or commercial rollout

From 2013 to the initial October 2018 legalization of non-medical cannabis, the rate of all cannabis cases in BC managed at DPIC increased by 20% annually (95% CI, 16%, 24%). This varied by cannabis form. Prior to their legalized sale in October 2018, the rate of cases involving inhalation of dried cannabis flower increased by 16% annually (11%, 22%) and the rate of cases involving consumption of oils and capsules increased by 29% annually (18%, 42%). Prior to their legal commercial rollout in January 2020, the rate of cases involving consumption of edibles increased by 30% annually (25%, 36%) and the rate of cases involving inhalation of concentrates increased by 42% annually (24%, 63%) (Fig. 2; Table 5).

**Table 5:** Interrupted time series model rate ratios for monthly counts of DPIC cannabis cases from 2013 to 2021 with annual provincial population as the model offset. For all cases, cases involving inhaled dried cannabis and ingestible oils and capsules, the intervention month was October 2018. For cases involving edibles and inhaled concentrates, the intervention month was January 2020. Age group strata with less than 100 cases were not modelled. Statistically significant rate ratios are bolded

	Case count	Pre-intervention annual rate ratio	Intervention level effect	Intervention slope effect	Post-intervention annual rate ratio
All cannabis cases	3989	**20% [16%, 24%]**	8% [− 8%, 27%]	− 1% [− 1%, − 1%]	**12% [6%, 19%]**
Cannabis edibles	1686	**30% [25%, 36%]**	3% [− 16%, 26%]	− 1% [− 2%, 1%]	**19% [4%, 36%]**
5 years and under	255	**52% [36%, 69%]**	11% [− 27%, 68%]	− 2% [− 4%, 1%]	23% [− 6%, 62%]
6 to 12 years	149	**57% [35%, 82%]**	12% [− 36%, 96%]	− 1% [− 4%, 2%]	40% [− 2%, 99%]
13 to 18 years	170	9% [− 2%, 22%]	**82% [3%, 224%]**	2% [− 2%, 6%]	32% [− 17%, 109%]
19 to 29 years	382	**39% [28%, 51%]**	− 23% [− 48%, 13%]	** − 3% [− 6%, − 1%]**	− 4% [− 28%, 28%]
30 + years	542	**33% [25%, 42%]**	− 9% [− 31%, 19%]	1% [− 1%, 3%]	**45% [17%, 81%]**
Inhaled dried cannabis	987	**16% [11%, 22%]**	− 12% [− 30%, 11%]	− 1% [− 2%, 1%]	8% [− 2%, 18%]
5 years and under	0	-	-	-	-
6 to 12 years	6	-	-	-	-
13 to 18 years	311	**17% [8%, 27%]**	** − 36% [− 58%, − 4%]**	− 1% [− 3%, 1%]	2% [− 15%, 23%]
19 to 29 years	324	**21% [10%, 32%]**	− 9% [− 42%, 44%]	1% [− 2%, 2%]	**21% [3%, 43%]**
30 + years	202	**16% [4%, 29%]**	35% [− 21%, 133%]	− 1% [− 2%, 2%]	13% [− 6%, 37%]
Ingestible cannabis oils and capsules	594	**29% [18%, 42%]**	26% [− 19%, 96%]	** − 5% [− 6%, − 3%]**	** − 14% [− 24%, − 2%]**
5 years and under	32	-	-	-	-
6 to 12 years	8	-	-	-	-
13 to 18 years	25	-	-	-	-
19 to 29 years	104	**23% [4%, 45%]**	48% [− 31%, 219%]	** − 5% [− 8%, − 2%]**	− 16% [− 37%, 10%]
30 + years	346	**20% [10%, 31%]**	45% [− 6%, 121%]	** − 5% [− 6%, − 3%]**	** − 13% [− 25%, − 1%]**
Inhaled cannabis concentrate	121	**42% [24%, 63%]**	− 24% [− 57%, 34%]	− 1% [− 5%, 3%]	26% [− 20%, 99%]
5 years and under	13	-	-	-	-
6 to 12 years	1	-	-	-	-
13 to 18 years	31	-	-	-	-
19 to 29 years	41	-	-	-	-
30 + years	20	-	-	-	-

### Legalization effect on time trends

Legalized sale did not have a significant effect on the monthly case rate in any models. The monthly rate of cases consuming oil and capsule products had a non-significant level increase by 26% (− 19%, 96%) upon legalized sale of these products in October 2018. The case rate then decreased to the end of the study period, but remained higher than the pre-intervention rate. Cases involving edible consumption, dried cannabis inhalation, and concentrate inhalation all continued to increase post-intervention, albeit at non-significant lower slopes compared to their pre-intervention trends (Fig. 2; Table 5).

### Age-stratified time trends

**Fig. 3:**
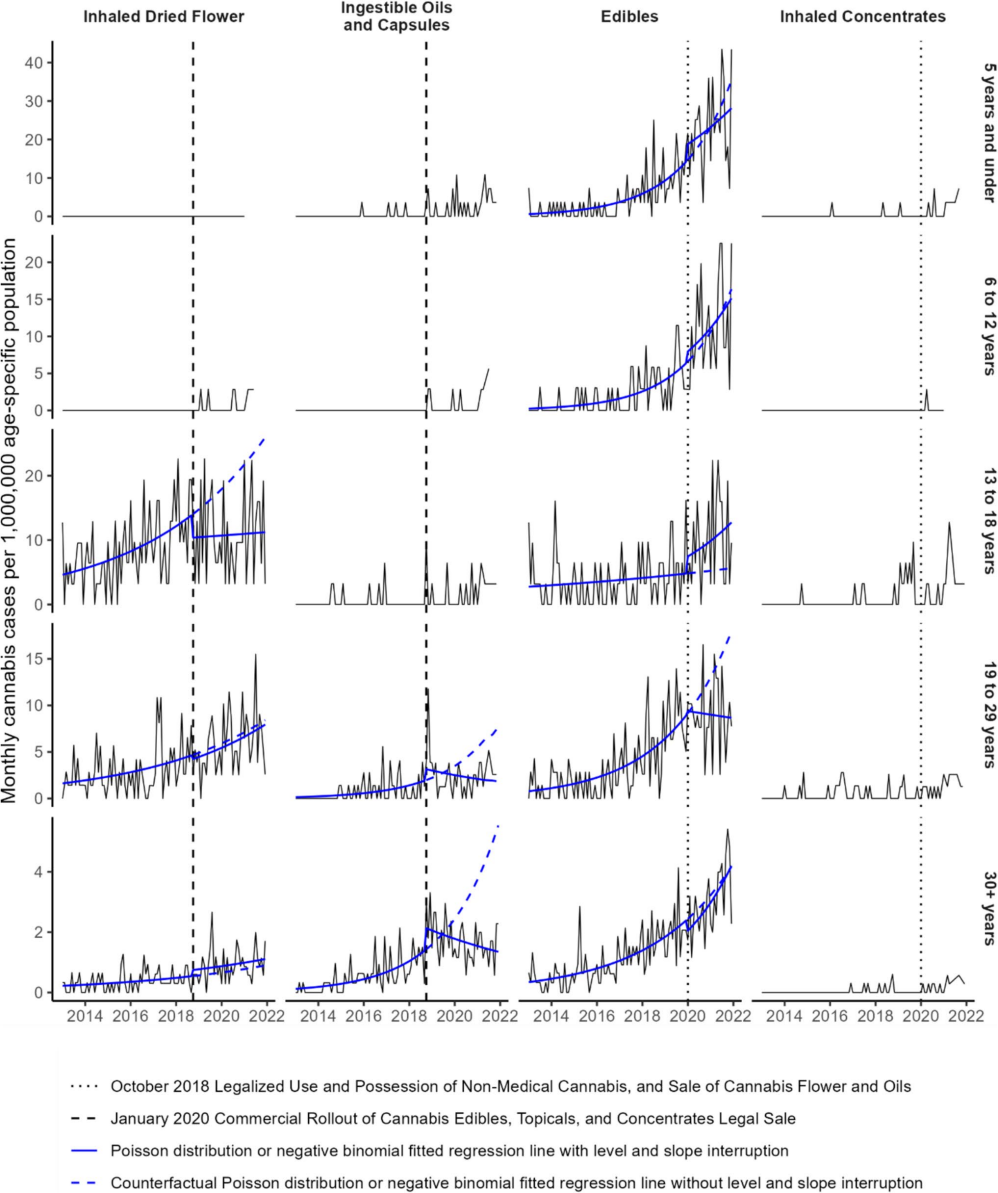
Monthly cannabis case rates per 1,000,000 population stratified by substance form and age group from 2013 through 2021. Negative binomial or Poisson distribution fitted regression lines were fit to each time series with a level and slope intervention at their respective month of legalized sale or commercial rollout

Most ingestible oil and capsule cases were among adults aged 19 and older (76%). These cases drove the decreasing trend post-intervention with a 16% decrease annually (− 37%, 10%) among young adults aged 19 to 29 and a 13% decrease annually (− 25%, − 1%) among adults aged 30 and older (Fig. 3; Table 5). Among the 65 ingestible oil and capsule cases involving children and teens aged 18 and younger, 54 (83%) were after legalization.

There were no cases of children aged 5 and under who had an inhalation exposure with dried cannabis and only 6 cases among children aged 6 to 12. All of these were after October 2018 legalization. Upon legalization, cases involving inhalation of dried cannabis among those aged 13–18 had a 36% decrease (− 58%, − 4%), while cases among those aged 30 and older had a 35% increase (− 21%, 133%).

The pre-intervention increase in edible cases was highest among children aged 5 and under and 6 to 12, with an annual increase of 52% (25%, 69%) and 57% (35%, 69%), respectively. Teens aged 13 to 18 had the smallest pre-intervention increase in edible cases at 9% (− 2%, 22%), but had an 82% level increase (3%, 224%) in January 2020, and an elevated slope post-intervention compared with pre-intervention. Post-intervention, the rate of cases among those aged 19 to 29 decreased by − 4% (− 28%, 28%) annually. The case rate for the age 30 plus group had an elevated slope post-intervention compared with pre-intervention, although this difference was not significant.

### Sensitivity analysis

The edible, inhaled dried cannabis and concentrate models were not sensitive to moving the intervention month 3 months forward or backward. The ingestible oil and capsule models were sensitive to the timing of the intervention month relative to the October through December 2018 spike in cases (Supplementary Material 2).

## Discussion

Prior to the legalization of non-medical cannabis use, possession, and restricted sale in 2018, the rate of calls made to DPIC involving cannabis exposure had been increasing over time. This aligns with the steady pre-legalization rise in self-reported cannabis use found in routine population surveys, as well as the pre-legalization rise in cannabis-related hospitalizations (Government of Canada, 2021a, 2022; Myran et al., 2023a). The reasons for this were likely multi-faceted. For example, the expansion of the medical marijuana market, legitimate or otherwise, in BC likely led to increased availability (Lough, 2015; Watson et al., 2019). Decreased stigma from impending legalization likely increased willingness to try cannabis and disclose use (Government of Canada, 2024).

Case rates involving edibles and cannabis inhalation continued to increase after legalization, albeit at a slower pace than pre-legalization. Among all groups, case rates of edible consumption among children aged 12 and under had the largest increase across the study period. Given that pediatric cannabis poisonings are most likely to occur at home (Varin et al., 2023), this rise is likely due to the overall increased availability and popularity of cannabis edibles among parents and other household members (Government of Canada, 2021b), thereby increasing the likelihood that a child could be exposed. There was a spike in ingestible oil and capsule cases upon legalization in October to December 2018, although this increase was not statistically significant. Wider availability due to legalization is a plausible explanation for this increase, given its timing and the high likelihood that Canadians acquire ingestible oil and capsule products from legal sources (Wadsworth et al., 2023). Overall, DPIC was supporting more calls involving cannabis exposure, regardless of its form, in 2021 than in 2013.

This analysis has some limitations to consider. First, classification of exposure intentionality and cannabis use history was determined from free-text case notes. Thus, availability was limited to information volunteered by the caller and documented by the SPI. We also sought information about product source, packaging, and storage, but these were unavailable in the vast majority of cases, and therefore not reported here. Such contextual information could have provided insights into pediatric cannabis poisonings in particular, as has been examined with sentinel surveillance hospitalization data (Varin et al., 2023). It could also allow for examination of whether exposure trends post-legalization were driven by legal regulated or illicit unregulated products. Second, SPIs document the biological sex of patients, but there is known misclassification between sex and gender in DPIC call records because this information is based on what the callers disclose or how they present over the phone. Third, personal identifiers were not consistently available in DPIC data, so we could not identify repeat subjects. We treated every case as independent in our analysis. Fourth, the relatively short post-intervention period provided less power for precise statistical estimates. We set the end of the study period to 2021 to allow for case reviews to be completed during 2022, which was essential for the valid stratification of cases by cannabis form (Table 6).

**Table 6:** Validation of substance form identification based on American Poison Center (APC) substance code and exposure route queries against substance form confirmed via case reviews, where applicable

Case-reviewed substance form	Case count	APC substance code query	Exposure route query	True-positive count (Sensitivity %)	False-negative count (%)	True-negative count (Specificity %)	False-positive count (%)
Edibles	1686	310,121 Marijuana: Edible Preparation	Ingestion	1424 (84%)	262 (16%)	2274 (99%)	29 (1%)
Inhaled dried flower	987	083000 Marijuana: Dried Plant	Direct inhalation	894 (91%)	93 (9%)	2789 (93%)	213 (7%)
Ingestible oils and capsules	594	310,122 Marijuana: Oral Capsule or Pill Preparation 310,124 Marijuana: Concentrated Extract	Ingestion	376 (63%)	218 (37%)	3324 (98%)	71 (2%)
Inhaled concentrates	121	310,033 eCigarettes: Marijuana Device Without Added Flavors 310,034 eCigarettes: Marijuana Device With Added Flavors 310,035 eCigarettes: Marijuana Liquid Without Added Flavors 310,036 eCigarettes: Marijuana Liquid With Added Flavors 310,096 eCigarettes: Marijuana Device Flavor Unknown 310,097 eCigarettes: Marijuana Liquid Flavor Unknown 310,124 Marijuana: Concentrated Extract	Direct inhalation	15 (12%)	106 (88%)	3867 (99.97%)	1 (0.03%)
Pharmaceutical cannabinoids	74	200,618 Marijuana: Pharmaceutical Preparation	Any except inhalation of fumes	41 (55%)	33 (45%)	3905 (99.7%)	10 (0.3%)
Ingestion of dried cannabis or paraphernalia	71	-	-	-	-	-	-
Second-hand cannabis smoke	66	083000 Marijuana: Dried Plant 200,617 Synthetic Cannabinoids, Analogs and Precursors 200,618 Marijuana: Pharmaceutical Preparation 310,033 eCigarettes: Marijuana Device Without Added Flavors 310,034 eCigarettes: Marijuana Device With Added Flavors 310,035 eCigarettes: Marijuana Liquid Without Added Flavors 310,036 eCigarettes: Marijuana Liquid With Added Flavors 310,096 eCigarettes: Marijuana Device Flavor Unknown 310,097 eCigarettes: Marijuana Liquid Flavor Unknown 310,124 Marijuana: Concentrated Extract	Inhalation of fumes	6 (33%)	12 (67%)	3971 (100%)	0 (0%)
Topicals	18	310,125 Marijuana: Topical Preparation	Dermal	7 (100%)	0 (0%)	3981 (99.97%)	1 (0.03%)
Synthetic cannabinoids	7	200,617 Synthetic Cannabinoids, Analogs and Precursors	Any except inhalation of fumes	44 (67%)	22 (33%)	3912 (99.7%)	11 (0.3%)
Other	6	-	-	-	-	-	-
Unknown	418	-	-	-	-	-	-

A final important limitation to note is that this interrupted time series analysis observed changes in the rate of calls to DPIC associated with, not caused by, specific time points. The Canadian cannabis market is and has been highly dynamic, including the illicit trade that has occurred for decades, the growth of non-medical retail since legalization, the legitimate medical dispensation, and the unregulated grey-area market in between all these sources. Exposure trends were likely impacted by several unmeasured external factors that did not align with the intervention periods we studied. These include the opening and closing of local retail stores, the availability of e-commerce and delivery, changes in pricing and availability of certain products, and marketing campaigns. In addition, the early 2020 commercial rollout of edible, topical, and concentrate products in the legal market approximately coincided with the beginning of the COVID-19 pandemic. This likely confounded DPIC call trends in complex ways. Reduced emergency department visits during the COVID-19 pandemic (Yao et al., 2023) led to reduced calls to DPIC from emergency care personnel overall. Conversely, some studies found an increase in cannabis use among Canadians during the COVID-19 pandemic due to stress, boredom, and social isolation (Newport et al., 2023). Future research may investigate cannabis call trends from Quebec’s poison centre as a comparator, given the province’s restricted commercial expansion of cannabis products in early 2020 compared with BC.

Regardless of the direct or indirect impacts of legalization, the rate of cannabis exposure events managed by DPIC was at an all-time high at the end of the study period in December 2021, and still trending upwards. These data highlight the importance of harm reduction measures in Canada’s legalized cannabis era. For one, promotion of low-risk use should continue in a thoughtful manner that does not stigmatize use (Steiner et al., 2019). These recommendations include being aware of product concentrations, choosing lower THC (tetrahydrocannabinol) products, beginning use at a lower dose of THC and increasing slowly, using in a safe set and setting, and avoiding use with other substances (Fischer et al., 2017). In this study, 2280 cases (57%) were among individuals aged 13 and older who used cannabis intentionally without self-harm intent. Low-risk use practices, such as the “start low, go slow” principle, may have been applicable to mitigate the unintended negative consequences for these users. Although the acute effects of cannabis intoxication can dissipate without intervention within a few hours, the symptoms and experience can be non-trivial, which can involve severe nausea, paranoia, and psychosis (Crocker et al., 2021). Heavy chronic use presents risks as well, with the potential to develop dependence, cannabis hyperemesis (i.e., a condition of chronic nausea and vomiting), and schizophrenia (Hoch et al., 2024).

Unintentional poisonings were another major group of cases found in this study (23%). THC doses as low as 1.7 mg/kg can illicit prolonged and severe effects (Pepin et al., 2023). While this dosage may be difficult to attain from accidental consumption by teens and adults, it can be relatively easy to achieve in infants and small children, with the most severe cases of pediatric cannabis poisonings resulting in coma or respiratory depression requiring intubation (Richards et al., 2017). Regulated edibles in Canada are limited to a maximum of 10 mg of THC per package, but some illicit products have been reported with more than 100 mg per small package (Government of Canada, 2023; CBC News, 2024). Beyond the promotion of safe storage practices to prevent unintentional poisonings for both children and adults, addressing the production, distribution, and sale of illicit unregulated cannabis products remains critical. These products often lack the safety measures of their legal regulated counterparts, such as child-resistant packaging, clear labelling and product warnings, and dosage limits.

## Conclusion

This study makes a unique contribution to the literature by examining cannabis exposure calls to a provincial poison centre based on the specific form of cannabis, and by applying a robust interrupted time series design to the analysis. Naturally, poison centres do not capture all problematic exposure events in a given jurisdiction. Several data sources should be leveraged to examine cannabis use and the impact of legalization in Canada (Hall et al., 2023). Crime and legal market sales data can provide indicators for social and economic impacts. Routine population surveys can measure shifting trends in behaviours and attitudes across the population. Hospitalization data can provide insight into severe poisonings and examination of specific health outcomes such as cannabis-induced psychosis, and in some cases, act as sentinel surveillance sites. Poison centres can also act as sentinels, providing detailed exposure information and capturing poisoning events that do not require additional health care contact. Poison centre data, among others, should continually be used to monitor trends in cannabis use and its effects.

## Contributions to knowledge

What does this study add to existing knowledge?

- This study leverages detailed exposure information available in British Columbia Drug and Poison Information Centre (DPIC) call records to characterize cannabis exposure events by the specific cannabis form.
- Stratified time series analyses by age and cannabis form revealed a spike in ingestible oil and capsule cases upon legalization among adults, and that the fastest rise in case rates overall were in pediatric edible consumption cases.
- This study supports findings from routine population surveys and analyses of hospitalization data which found that cannabis use and associated harms have been increasing since before legalization of non-medical cannabis in Canada.

What are the key implications for public health interventions, practice, or policy?

- The increasing frequency of pediatric and unintentional adult cannabis poisonings highlights the importance of safe storage practices, child-resistant packaging, clear labelling, and the control of illicit products.
- There is a nascent rise in exposure events involving cannabis concentrate inhalation. These are relatively newer products on the market and should continue to be monitored.
- Poison centre data allow for the surveillance of events that do not require further medical attention, which accounted for 60% of cannabis cases managed at DPIC from 2013 to 2021.

## Supplementary Information

Below is the link to the electronic supplementary material.

Supplementary file1 (PDF 106 KB)

Supplementary file2 (PDF 48 KB)

## Data Availability

Not applicable.

## References

[CR1] Allaf, S., Lim, J. S., Buckley, N. A., & Cairns, R. (2023). The impact of cannabis legalization and decriminalization on acute poisoning: A systematic review. *Addiction,**118*(12), 2252–2274. 10.1111/add.16280 37496145 10.1111/add.16280 PMC10952774

[CR2] CBC News. (2024). *120,000 cannabis-laced candy edibles seized from Vancouver Island dispensaries*. https://www.cbc.ca/news/canada/british-columbia/cannabis-candies-1.7367176

[CR3] Crocker, C. E., Carter, A. J. E., Emsley, J. G., Magee, K., Atkinson, P., & Tibbo, P. G. (2021). When cannabis use goes wrong: Mental health side effects of cannabis use that present to emergency services. *Frontiers in Psychiatry,**12*, 640222. 10.3389/fpsyt.2021.640222 33658953 10.3389/fpsyt.2021.640222 PMC7917124

[CR4] Durigon, M., Elliott, C., Purssell, R., & Kosatsky, T. (2013). Canadian poison control centres: Preliminary assessment of their potential as a resource for public health surveillance. *Clinical Toxicology,**51*(9), 886–891. 10.3109/15563650.2013.841182 24134535 10.3109/15563650.2013.841182

[CR5] Fischer, B., Russell, C., Sabioni, P., van den Brink, W., Le Foll, B., Hall, W., Rehm, J., & Room, R. (2017). Lower-risk cannabis use guidelines: A comprehensive update of evidence and recommendations. *American Journal of Public Health,**107*(8), e1–e12. 10.2105/AJPH.2017.303818 28644037 10.2105/AJPH.2017.303818 PMC5508136

[CR6] Gelman, A., & Hill, J. (2007). Poisson regression, exposure, and overdispersion. In *Data**analysis using regression and multilevel/hierarchical models* (pp. 110–116).

[CR7] Government of British Columbia. (2019). *Cannabis edibles, extracts and**topicals available soon in B.C. | BC Gov**News*. https://news.gov.bc.ca/releases/2019AG0132-002451

[CR8] Government of British Columbia. (2021). *Population**estimates—Province of British Columbia*. Province of British Columbia. https://www2.gov.bc.ca/gov/content/data/statistics/people-population-community/population/population-estimates

[CR9] Government of Canada. (2018). *Cannabis**legalization and regulation*. https://www.justice.gc.ca/eng/cj-jp/cannabis/

[CR10] Government of Canada. (2021a). *Canadian Alcohol and Drugs Survey* [Surveys]. https://www.canada.ca/en/health-canada/services/canadian-alcohol-drugs-survey.html

[CR11] Government of Canada. (2021b). *Looking back from 2020, how cannabis use and related**behaviours**changed in Canada*. Statistics Canada. https://www150.statcan.gc.ca/n1/pub/82-003-x/2021004/article/00001-eng.htm 10.25318/82-003-x202100400001-eng 33881274

[CR12] Government of Canada. (2022). *Canadian Cannabis Survey 2022: Summary* [Surveys]. https://www.canada.ca/en/health-canada/services/drugs-medication/cannabis/research-data/canadian-cannabis-survey-2022-summary.html

[CR13] Government of Canada. (2023). *Accidental ingestion of illegal “copycat” edible cannabis products causing serious harm to children—Recalls, advisories and safety alerts – Canada.ca*. Government of Canada, Health Canada, Communications and Public Affairs Branch. https://recalls-rappels.canada.ca/en/alert-recall/accidental-ingestion-illegal-copycat-edible-cannabis-products-causing-serious-harm

[CR14] Government of Canada. (2024). *Canadian Cannabis Survey 2023: Summary* [Surveys]. https://www.canada.ca/en/health-canada/services/drugs-medication/cannabis/research-data/canadian-cannabis-survey-2023-summary.html

[CR15] Hall, W., Stjepanović, D., Dawson, D., & Leung, J. (2023). The implementation and public health impacts of cannabis legalization in Canada: A systematic review. *Addiction,**118*(11), 2062–2072. 10.1111/add.16274 37380613 10.1111/add.16274 PMC10953418

[CR16] Hoch, E., Volkow, N. D., Friemel, C. M., Lorenzetti, V., Freeman, T. P., & Hall, W. (2024). Cannabis, cannabinoids and health: A review of evidence on risks and medical benefits. *European Archives of Psychiatry and Clinical Neuroscience*. 10.1007/s00406-024-01880-2 39299947 10.1007/s00406-024-01880-2 PMC11910417

[CR17] Lough, S. (2015). Dispensaries: The Wild West of Vancouver. *CMAJ,**187*(11), E345–E346. 10.1503/cmaj.109-5116 26195580 10.1503/cmaj.109-5116 PMC4527928

[CR18] Myran, D. T., Gaudreault, A., Konikoff, L., Talarico, R., & Liccardo Pacula, R. (2023a). Changes in cannabis-attributable hospitalizations following nonmedical cannabis legalization in Canada. *JAMA Network Open,**6*(10), e2336113. 10.1001/jamanetworkopen.2023.36113 37796504 10.1001/jamanetworkopen.2023.36113 PMC10556968

[CR19] Myran, D. T., Tanuseputro, P., Auger, N., Konikoff, L., Talarico, R., & Finkelstein, Y. (2023b). Pediatric hospitalizations for unintentional cannabis poisonings and all-cause poisonings associated with edible cannabis product legalization and sales in Canada. *JAMA Health Forum,**4*(1), e225041. 10.1001/jamahealthforum.2022.5041 36637814 10.1001/jamahealthforum.2022.5041 PMC9857209

[CR20] Newport, K., Bishop, L., Donnan, J., Pal, S., & Najafizada, M. (2023). The COVID-19 pandemic and cannabis use in Canada—A scoping review. *Journal of Cannabis Research,**5*, 31. 10.1186/s42238-023-00196-7 37525289 10.1186/s42238-023-00196-7 PMC10388476

[CR21] Pepin, L. C., Simon, M. W., Banerji, S., Leonard, J., Hoyte, C. O., & Wang, G. S. (2023). Toxic tetrahydrocannabinol (THC) dose in pediatric cannabis edible ingestions. *Pediatrics,**152*(3), e2023061374. 10.1542/peds.2023-061374 37635689 10.1542/peds.2023-061374

[CR22] R Core Team. (2021). *R: A language and environment for statistical computing.**R Foundation for Statistical Computing*. https://www.r-project.org/

[CR23] Rahim, T. (2019). *Cannabis poisoning in BC: Insights from poison control**centre**calls | National Collaborating Centre for Environmental Health | NCCEH - CCSNE*. https://ncceh.ca/resources/blog/cannabis-poisoning-bc-insights-poison-control-centre-calls

[CR24] Richards, J. R., Smith, N. E., & Moulin, A. K. (2017). Unintentional cannabis ingestion in children: A systematic review. *The Journal of Pediatrics,**190*, 142–152. 10.1016/j.jpeds.2017.07.005 28888560 10.1016/j.jpeds.2017.07.005

[CR25] Steiner, L., Nicol, A.-M., & Eykelbosh, A. (2019). How we talk about “Pot” matters: Strategies for improved cannabis risk communication. *Environmental Health Review,**62*(1), 8–13. 10.5864/d2019-005

[CR26] Varin, M., Champagne, A., Venugopal, J., Li, L., McFaull, S. R., Thompson, W., Toigo, S., Graham, E., & Lowe, A.-M. (2023). Trends in cannabis-related emergency department visits and hospitalizations among children aged 0–11 years in Canada from 2015 to 2021: Spotlight on cannabis edibles. *BMC Public Health,**23*(1), 2067. 10.1186/s12889-023-16987-9 37872564 10.1186/s12889-023-16987-9 PMC10591397

[CR27] Wadsworth, E., Rynard, V., Driezen, P., Freeman, T. P., Rychert, M., Wilkins, C., Hall, W., Gabrys, R., & Hammond, D. (2023). Legal sourcing of ten cannabis products in the Canadian cannabis market, 2019–2021: A repeat cross-sectional study. *Harm Reduction Journal,**20*(1), 19. 10.1186/s12954-023-00753-6 36803833 10.1186/s12954-023-00753-6 PMC9936931

[CR28] Watson, T. M., Hyshka, E., Bonato, S., & Rueda, S. (2019). Early-stage cannabis regulatory policy planning across Canada’s four largest provinces: A descriptive overview. *Substance Use & Misuse,**54*(10), 1691–1704. 10.1080/10826084.2019.1608249 31076006 10.1080/10826084.2019.1608249

[CR29] Yao, J., Irvine, M. A., Klaver, B., Zandy, M., Dheri, A. K., Grafstein, E., & Smolina, K. (2023). Changes in emergency department use in British Columbia, Canada, during the first 3 years of the COVID-19 pandemic. *Canadian Medical Association Journal,**195*(34), E1141–E1150. 10.1503/cmaj.221516 37669788 10.1503/cmaj.221516 PMC10480001

