# Supplementary material for: Impact of legalization on cannabis exposure calls to the British Columbia Poison Control Centre
Source: Can J Public Health. 2025 Apr 7;117(1):151–66. doi: 10.17269/s41997-025-01022-8 (PMC12992831; doi:10.17269/s41997-025-01022-8)
Supplement: Supplementary file 1 — Supplementary file1 (PDF 106 KB) [file 41997_2025_1022_MOESM1_ESM.pdf]

Period	Substance Form	Age Group	Kruskal-Wallis Test Statistic	P-Value
Month	All cases	All ages	2.64	0.99
Month	Cannabis edibles	All ages	4.7	0.94
Month	Cannabis edibles	5 years and under	9.1	0.61
Month	Cannabis edibles	6 to 12 years	12.55	0.32
Month	Cannabis edibles	13 to 18 years	11.73	0.38
Month	Cannabis edibles	19 to 29 years	5.76	0.89
Month	Cannabis edibles	30+ years	8.08	0.71
Month	Inhaled dried cannabis	All ages	4.08	0.97
Month	Inhaled dried cannabis	13 to 18 years	3.35	0.99
Month	Inhaled dried cannabis	19 to 29 years	10.58	0.48
Month	Inhaled dried cannabis	30+ years	12.28	0.34
Month	Ingestible oils and capsules	All ages	5.24	0.92
Month	Ingestible oils and capsules	19 to 29 years	10.15	0.52
Month	Ingestible oils and capsules	30+ years	8.89	0.63
Month	Inhaled concentrates	All ages	13.21	0.28
Quarter	All cases	All ages	0.39	0.94
Quarter	Cannabis edibles	All ages	0.28	0.96
Quarter	Cannabis edibles	5 years and under	0.06	1
Quarter	Cannabis edibles	6 to 12 years	0.19	0.98
Quarter	Cannabis edibles	13 to 18 years	4.56	0.21
Quarter	Cannabis edibles	19 to 29 years	0.28	0.96
Quarter	Cannabis edibles	30+ years	0.58	0.9
Quarter	Inhaled dried cannabis	All ages	1.15	0.77
Quarter	Inhaled dried cannabis	13 to 18 years	0.38	0.95
Quarter	Inhaled dried cannabis	19 to 29 years	2.43	0.49
Quarter	Inhaled dried cannabis	30+ years	2.28	0.52
Quarter	Ingestible oils and capsules	All ages	0.65	0.88
Quarter	Ingestible oils and capsules	19 to 29 years	2.16	0.54
Quarter	Ingestible oils and capsules	30+ years	0.97	0.81
Quarter	Inhaled concentrates	All ages	4.76	0.19
