# Supplementary figures and images for: Impact of legalization on cannabis exposure calls to the British Columbia Poison Control Centre
Source: Can J Public Health. 2025 Apr 7;117(1):151–66. doi: 10.17269/s41997-025-01022-8 (PMC12992831; doi:10.17269/s41997-025-01022-8)

# Cannabis Edibles

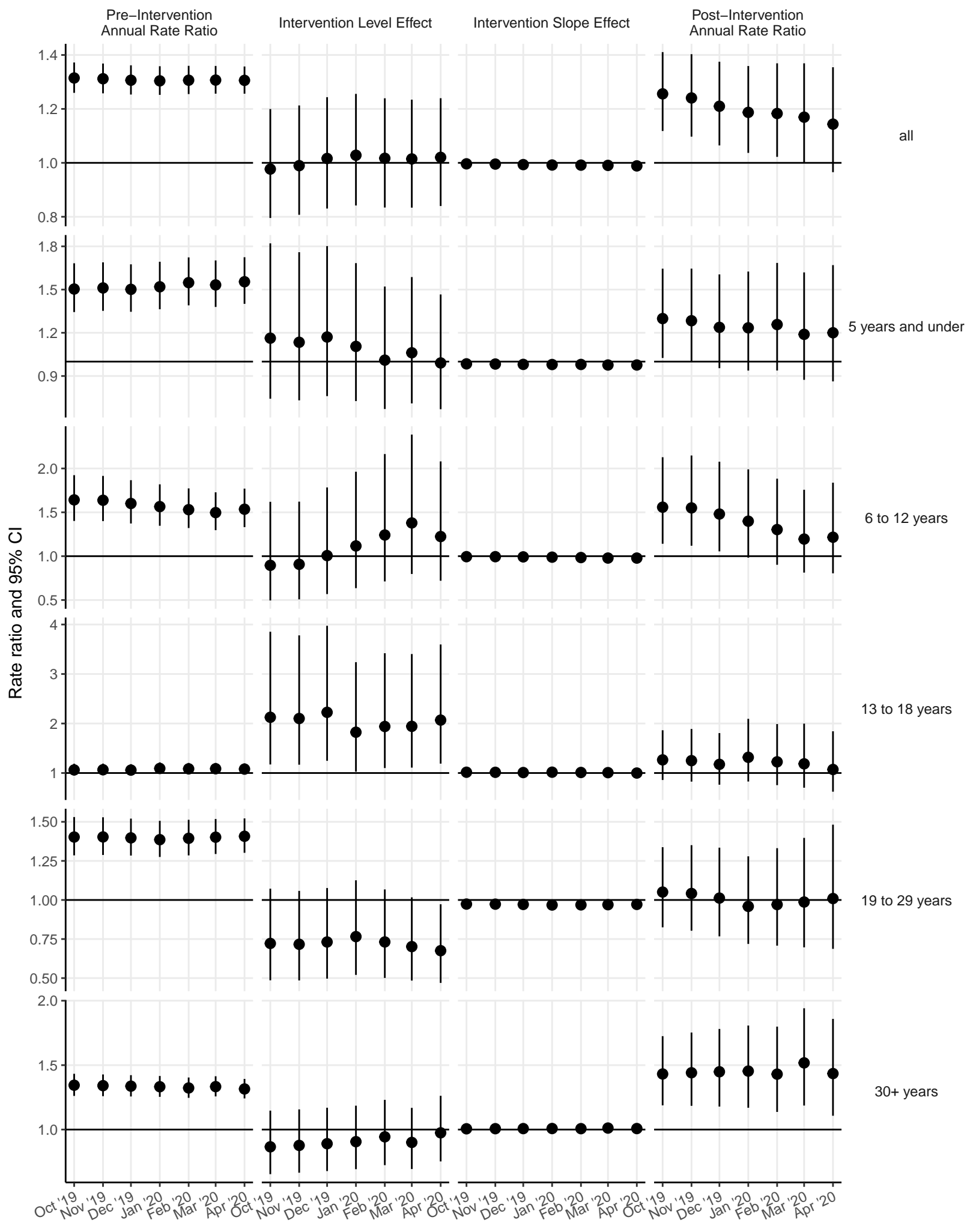

# Inhaled Dried Flower

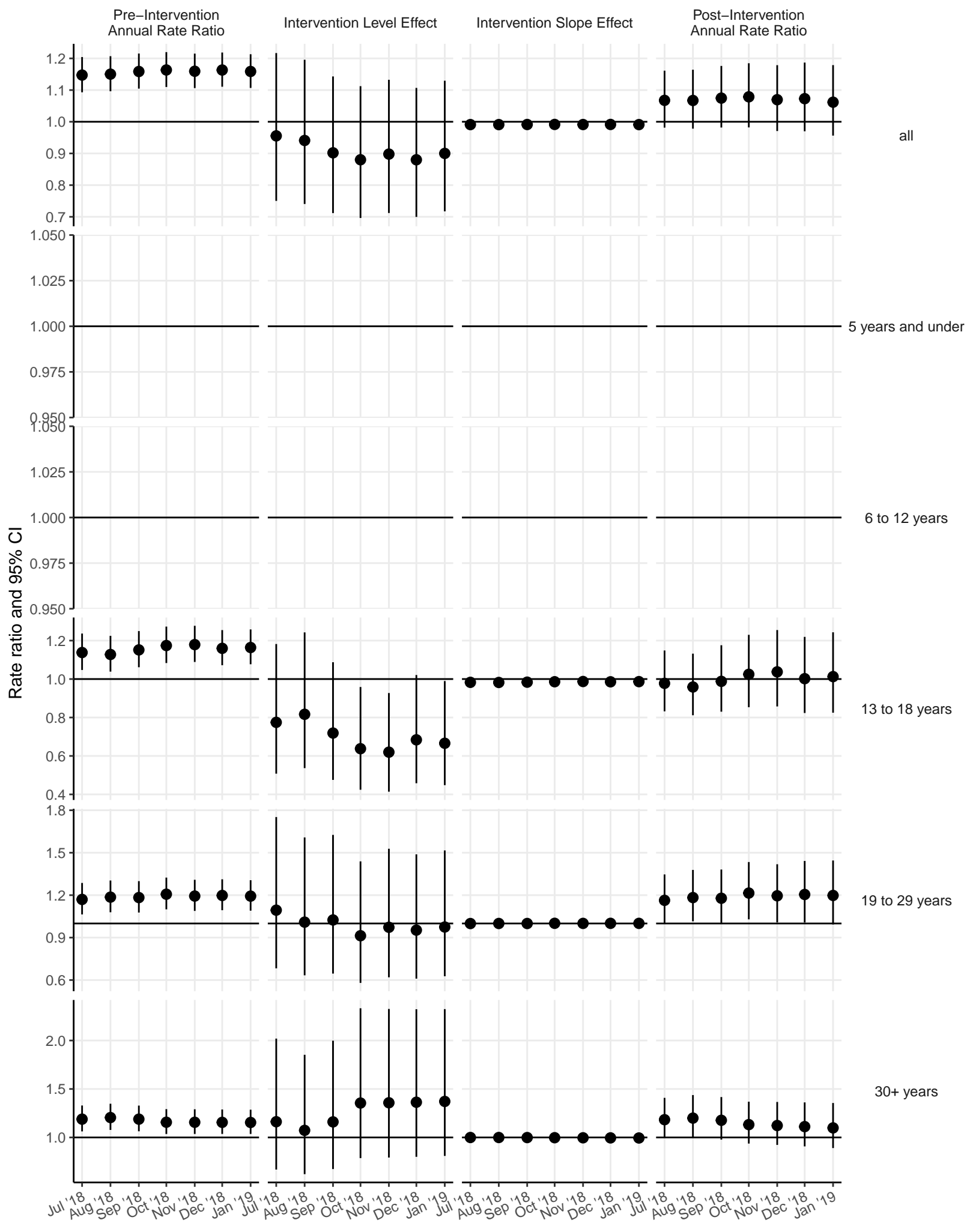

# Ingestible Oils and Capsules

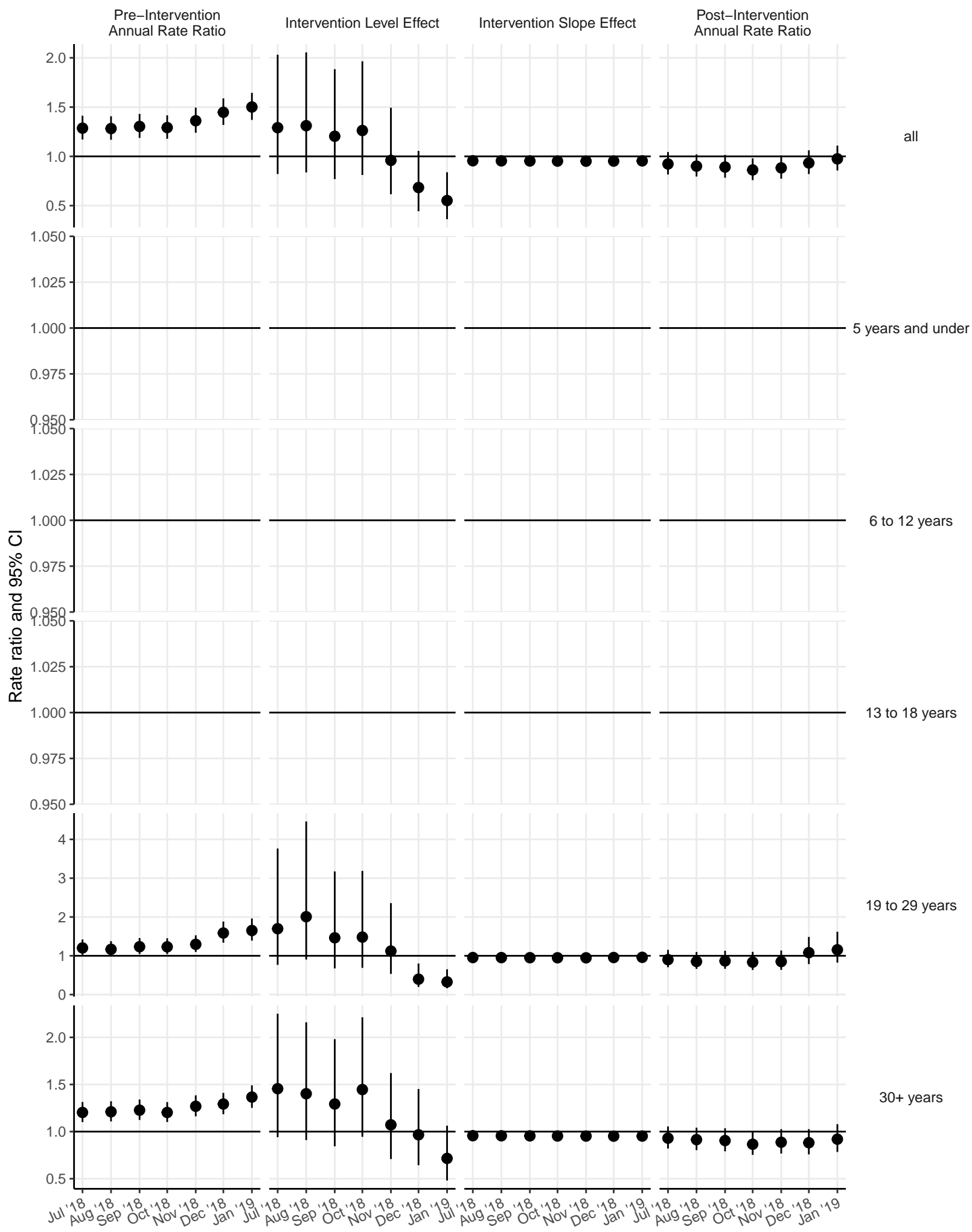

# Inhaled Concentrate

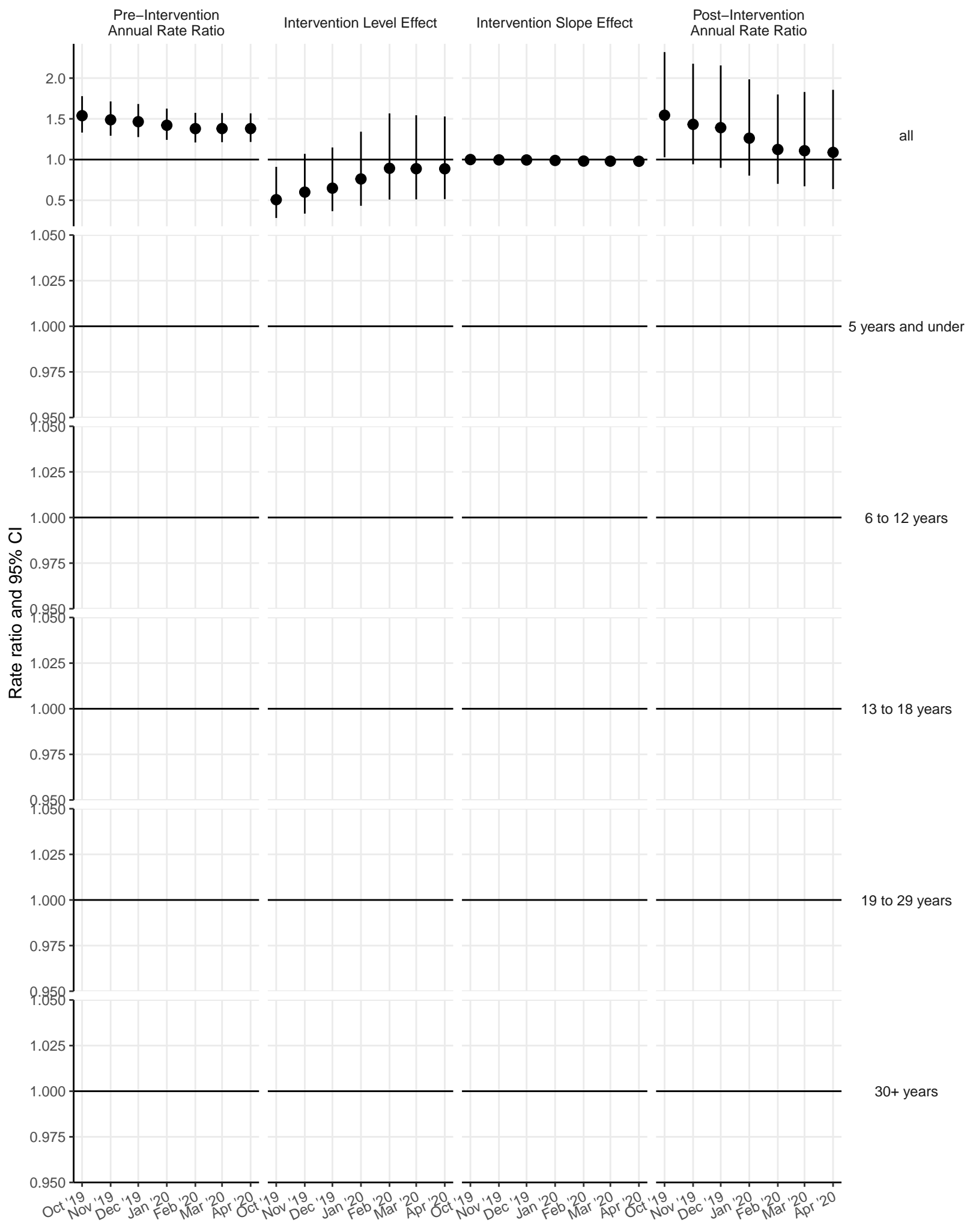

Supplement: Supplementary file 2 — Supplementary file2 (PDF 48 KB) [file 41997_2025_1022_MOESM2_ESM.pdf]
